# College and Hospital Notes

**Published:** 1901-06

**Authors:** 


### COLLEGE AND HOSPITAL NOTES.

The Albany Hospital, which was practically completed last year, has undergone many needed improvements lately, so that it is now one of the best equipped and most superb of all our modern hospitals. Its operating room is a model of perfection; its walls and ceiling are of white glazed tile, its window and door cases of marble, its floor of tile and its operating table is of the most modern and convenient construction. The total cost of the hospital exclusive of the land is $294,918.38. The institution is a credit to the men who have supervised and carried to completion this splendid structure.

---

The Dental Department U. of B. graduated a large class May 14, 1901. The exercises were held at the Star Theatre and the address to the graduates was delivered by Dr. Archibald Dann, of Rochester.

---

The State Hospital for the care of persons suffering from incipient tuberculosis is now an assured fact. Governor Odell has signed the bill appropriating $100,000, to be expended in building it, which will enable the managers to put the scheme to an adequate test.

---

The Niagara Falls Memorial Hospital graduated four nurses from its training school May 14, 1901. Dr. F. Park Lewis, of Buffalo, addressed the graduates, who were the Misses Margaret Catharine

Madigan, Florence A. Manley, Cecilia Brown, and Josephine H. Eddy. A reception followed the exercises.

---

THE Buffalo Homeopathic Hospital has appointed the following staff for the current year: Consulting physicians, Alexander T. Bull, Henry Baethig, H. A. Foster, John Miller, A. M. Curtiss, D. B. Stumpf, P. A. McCrea. Attending physicians, E. P. Hussey, J. T. Cook, B. J. Maycock, T. J. Martin, E. A. Fisher. Attending Surgeons, H. C. Frost, G. T. Moseley, D. G. Wilcox, W. H. Marcy. Obstetricians, G. R. Stearns, J. S. Halbert, Jessie Shepard. Ophthalmic surgeon, F. Park Lewis. Pathologist, George R. Critchlow. Laryngologist, Frederick D. Lewis.

The officers are: President, Dr. Burt J. Maycock; first vice-president, Dr. Henry Baethig; second vice-president, Dr. Elisha P. Hussey; secretary, Dr. Joseph T. Cook.

